# Synthesis and Luminescent Properties of Eu^3+^-Doped Complex Borosilicate Glasses

**DOI:** 10.3390/molecules31061000

**Published:** 2026-03-16

**Authors:** Aneliya Yordanova, Margarita Milanova, Lyubomir Aleksandrov, Reni Iordanova, Petia Petrova

**Affiliations:** 1 Institute of General and Inorganic Chemistry, Bulgarian Academy of Sciences, G. Bonchev Str., Bld. 11, 1113 Sofia, Bulgaria; a.yordanova@svr.igic.bas.bg (A.Y.); lubomirivov@gmail.com (L.A.); reni@svr.igic.bas.bg (R.I.); 2 National Centre of Excellence Mechatronics and Clean Technologies, 8 bul., Kl. Ohridski, 1756 Sofia, Bulgaria; 3 Institute of Optical Materials and Technologies “Acad. Jordan Malinowski”, Bulgarian Academy of Sciences, Blvd. Akad. G. Bonchev Str., Bld. 109, 1113 Sofia, Bulgaria; petia@iomt.bas.bg

**Keywords:** glass, rare earths, photoluminescence, density

## Abstract

Glasses with compositions (52.5 − x/2)B_2_O_3_:(12.5 − x/2)SiO_2_:25La_2_O_3_:5ZnO:5CaO:0.5Eu_2_O_3_:xWO_3_, x = 0, 2.5, 5, 7.5, 10, 20 (mol%) were prepared by conventional melt-quenching method and investigated by X-ray diffraction analysis, DSC analysis, DR-UV-Vis spectroscopy and photoluminescence spectroscopy. Physical parameters like density, molar volume, oxygen molar volume and oxygen packing density were also determined. Their values, as well as DR-UV-Vis spectroscopy results, indicate that the tungstate ions incorporate into the base borosilicate glass as tetrahedral WO_4_ and octahedral WO_6_ groups. With increasing WO_3_ content over 5 mol%, WO_6_ units are progressively linked to each other by W-O-W bonds, leading to the formation of a more connected and homogeneous glass network. Glasses are characterized by a high glass transition temperature (over 650 °C) and good thermal stability. The emission intensity of the Eu^3+^ ion increases with the introduction of WO_3_ due to the occurrence of non-radiative energy transfer from the tungstate groups to the active ion. The most intense luminescence peak observed at 612 nm suggests that the glasses are potential materials for red emission.

## 1. Introduction

Over recent decades, the development and analysis of fluorescent materials doped with rare earth ions have garnered significant interest within the field of optoelectronics research. This growing attention stems from their wide array of applications in the creation and advancement of novel optical materials. Glasses, in particular, stand out as some of the most thoroughly studied engineering materials due to their versatility and adaptability achieved through composition modification. Additionally, they hold immense potential in innovations for optical communication and solid-state laser technologies [1]. Among the various types of glasses, borosilicate glasses have captured researchers’ attention because of their remarkable properties. These include high chemical resistance, a high crystallization ability, lower thermal expansion, elevated softening temperature, and excellent mechanical strength. They are cost-effective and readily accessible. Such properties pave the way for extensive industrial applications in areas such as display technologies, solar energy systems, and MEMs technology.

In advanced technologies, trivalent rare earth ions play a pivotal role as active constituents in numerous optical materials. Their importance is owed to the presence of multiple absorption and emission bands that result from transitions between distinct energy levels. Notably, trivalent europium (Eu^3+^) ions stand out as effective spectroscopic probes due to their simple energy-level structure, which features a non-degenerate ^7^F_0_ ground state and ^5^D_0_ excited state. This makes Eu^3+^ ions instrumental in studying the structure and chemical bonding nature within host matrices. Moreover, the strong ^5^D_0_–^7^F_2_ electronic transition exhibited by these ions establishes them as efficient activators for generating intense red emission, particularly suited for display devices [1].

Borosilicate glasses doped with Eu_2_O_3_ oxide have been extensively studied. For example, Bi-containing borosilicate glasses doped with different amounts of Eu_3_O_2_ (1–4 mol%) were synthesized. The influence of the concentration of Eu_2_O_3_ on the physical, optical and luminescent properties of the glasses was studied. It was found that the glasses show the strongest emission at a wavelength of 613 nm and when excited by 465 nm. The color of the emission is reddish-orange. The optimal concentration of Eu_2_O_3_ in these glasses, at which the highest emission intensity is achieved, is 4.0 mol% [2]. Eu^3+^-doped glasses with the composition 74.5B_2_O_3_ + 10SiO_2_ + 5MgO + R + 0.5Eu_2_O_3_ [R = 10 (Li_2_O/Na_2_O/K_2_O)] are considered to be potential candidates for red lasers, as well as for red color centers in displays [1]. Zinc–borosilicate glasses (with high ZnO content) doped with different amounts of Eu_2_O_3_ (0.2, 0.5, 1, 1.5, 2 mol%) were obtained. The glasses are characterized by intense red emission upon excitation with a wavelength of 395 nm. “Quenching” of the luminescence is observed at a concentration of Eu_2_O_3_ above 1 mol% [3,4]. Thermally stable borosilicate glasses doped with Eu_2_O_3_ with the composition 35B_2_O_3_.20SiO_2_.(15 − x)Al_2_O_3_.15ZnO.15Na_2_CO_3_.xEu_2_O_3_ (x = 0.5 ÷ 2.5 mol%) were synthesized. These glasses showed red emission, the intensity of which increased with increasing concentration of Eu^3+^ ions up to 2.5 mol% [5]. The effect of changing the concentration of B_2_O_3_ and Al_2_O_3_ in the composition of the glass Na_2_O-Gd_2_O_3_-B_2_O_3_-SiO_2_-Al_2_O_3_-Eu_2_O_3_ on the luminescent properties of the incorporated Eu^3+^ ions was studied. It was shown that with the addition of Al_2_O_3_, a [BO_4_] → [BO_3_] transformation occurs. The amorphous network becomes ‘‘loose’’, resulting in an increase in the space around the rare earth ions embedded in the glass matrix, an increase in their quenching concentration, and an improvement in the emission intensity of Eu^3+^ [6]. Physical, optical, and luminescent properties of Eu^3+^-doped potassium borosilicate glasses (KBSi:Eu^3+^) have been studied. The density and molar volume of the glasses increase with increasing Eu_2_O_3_ oxide content. After excitation with 394 nm, KBSi:Eu^3+^ glasses emit highly intense reddish-orange light and could be applied in various photonic devices such as solid-state lasers and light-emitting diodes [7]. The optical and luminescent properties of Eu^3+^-doped glasses with the composition (55 − x)B_2_O_3_:10SiO_2_:25Y_2_O_3_:10CaO:xEu_2_O_3_, x = 0 ÷ 2.5 mol%, were studied [8]. The color coordinates of the glasses were found to be in the red region. The resulting glass was a potential candidate for red laser emission at 613 nm. Sm^3+^/Eu^3+^ co-doped thermally stable zinc–alumino-borosilicate glasses and alkaline-earth–alumino-borosilicate glasses were investigated. They were shown to be suitable candidates for application as red components of white-light-emitting diodes [9,10]. Dy^3+^/Eu^3+^-co-doped luminescent glasses SiO_2_-B_2_O_3_-ZnO-La_2_O_3_-BaO (SBZLBA) were synthesized. When excited at 386 nm, the glasses exhibit three distinct emission regions: blue, yellow, and red, allowing for color-tunable luminescence ranging from cool white to neutral white and ultimately warm white by varying the excitation wavelength and Eu^3+^ doping concentration. Results indicate that Dy^3+^/Eu^3+^-co-doped SBZLBa glasses are promising materials for white-light-emitting devices [11]. Dy^3+^/Eu^3+^-co-doped white-light-emitting CaO-B_2_O_3_-SiO_2_ glasses have been obtained. The glasses exhibit good thermal stability, and the luminescence color can be tuned by controlling the relative concentrations of Dy^3+^ and Eu^3+^ ions and the excitation wavelength. White light was achieved upon excitation at 387 nm when the concentrations of Dy^3+^ and Eu^3+^ were 4% and 2%, respectively [12].

It should be noted that in many cases the emission intensity of Eu^3+^ ions is higher when they are embedded in matrices containing tungsten oxide, compared to their intensity in other matrices. The significance of tungstates is determined by the occurrence of non-radiative charge transfer from WO_n_ groups to the active Re^3+^ (Nd, Sm, Eu, Tb, Dy) ion, which leads to an increase in the intensity and efficiency of the emission. There are very few studies in the literature on borosilicate glasses containing tungsten oxide, for example, compositions B_2_O_3_-SiO_2_-ZnO-Na_2_O-WO_3_ [13] and 20B_2_O_3_–10SiO_2_–10CaO-(60 − x)Bi_2_O_3_/xWO_3_, x = 0 to 20 wt% [14], and the research on these composites is mainly devoted to structural properties. Although the properties of borosilicate glasses doped with Eu^3+^ are widely investigated [2,3,4,5,6,7,8,9,10,11,12], their complex interaction with different WO_3_ doping levels has not yet been studied.

This work aims to investigate the influence of WO_3_ on the physical parameters and luminescent characteristics of Eu^3+^-doped (52.5 − x/2)B_2_O_3_:(12.5 − x/2)SiO_2_:25La_2_O_3_:5ZnO:5CaO:0.5Eu_2_O_3_:xWO_3_, x = 0, 2.5, 5, 7.5, 10 and 20 mol% glasses.

## 2. Results and Discussion

### 2.1. XRD Data and Thermal Analysis

**Figure 1:**
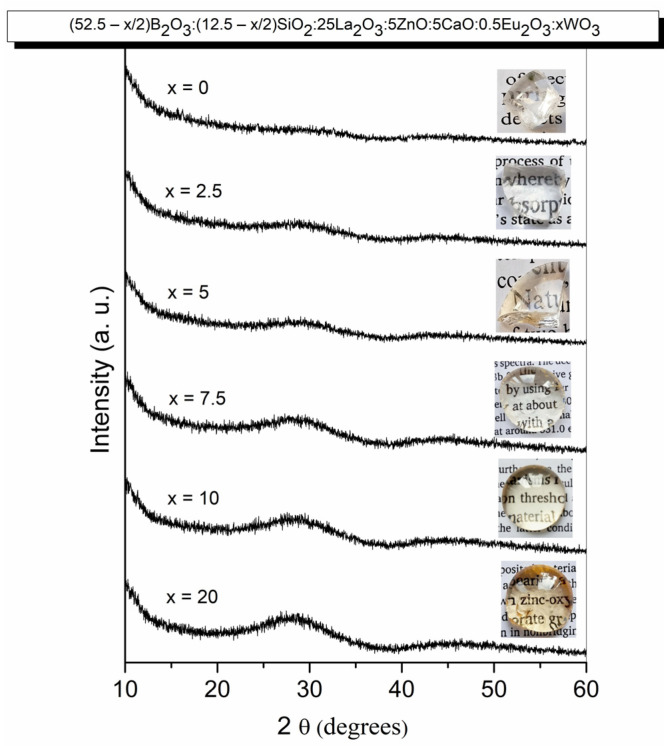
XRD patterns and photographs (insets) of investigated glasses.

The amorphous nature of the prepared materials was confirmed by X-ray diffraction analysis. Typical diffraction patterns of the glasses obtained are shown in Figure 1. The photographic images (insets, Figure 1) show that transparent bulk glass specimens were obtained.

**Figure 2:**
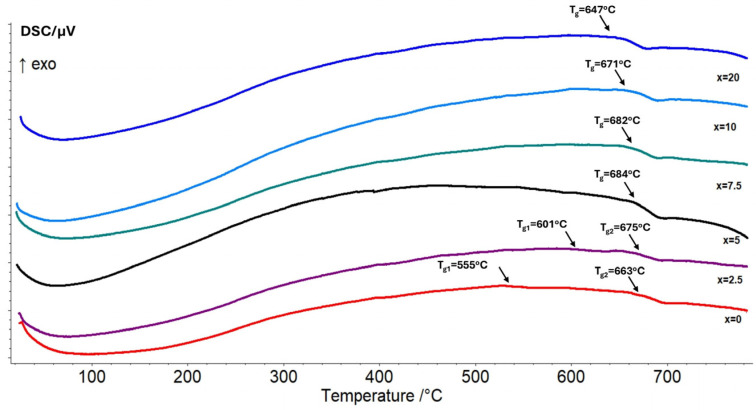
DSC curves of (52.5 − x/2)B_2_O_3_:(12.5 − x/2)SiO_2_:25La_2_O_3_:5ZnO:5CaO:0.5Eu_2_O_3_:xWO_3_, x = 0, 2.5, 5, 7.5, 10 and 20 mol% glasses.

The obtained glasses were also investigated using DSC analysis in order to obtain information on their thermal parameters and on some structural changes that take place due to compositional changes. Figure 2 compares the DSC curves of the glasses investigated in this work. The glass transition temperature, T_g_, has been determined (Table 1), since it is connected with both the strength of inter-atomic bonds and glass network connectivity. A higher T_g_ corresponds to a more rigid structure, whereas the glasses with a loose-packed structure have a lower T_g_ [15]. For glasses x = 0 and x = 2.5, two glass transition temperatures are obtained due to the formation of two amorphous phases because of liquid phase separation during the melting process. With further increase in WO_3_ content, one T_g_ is observed, indicating that liquid phase separation has been overcome. Hence, the addition of more than 2.5 mol% tungsten oxide into the base glass leads to the formation of a homogeneous amorphous network. It is seen that T_g_ values decrease for glasses containing more than 5 mol%WO_3_ (from 684 to 647 °C). The observed reduction in T_g_ is a result of the replacement of strong Si-O and B-O bonds with weaker W-O bonds [16].

### 2.2. DR-UV–Vis Spectra

**Figure 3:**
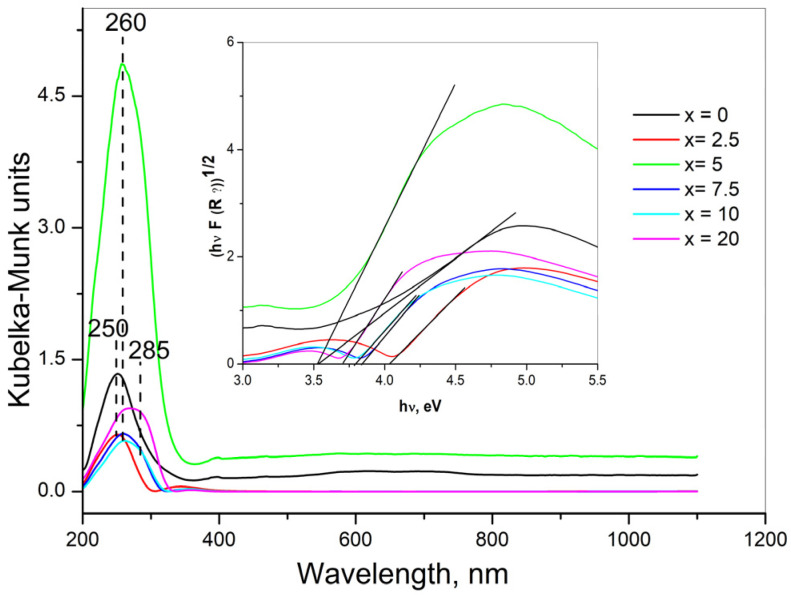
UV–Vis optical spectra and Tauk plots (the insets) of (52.5 − x/2)B_2_O_3_:(12.5 − x/2)SiO_2_:25La_2_O_3_:5ZnO:5CaO:0.5Eu_2_O_3_:xWO_3_, x = 0, 2.5, 5, 7.5, 10 and 20 mol% glasses.

Diffuse Reflectance UV-Vis Spectroscopy (DRS) was also applied for the characterization of the prepared materials. Figure 3 shows the diffuse reflectance spectra of the glasses obtained.

In the spectrum of WO_3_-free glass, one symmetrical band at 250 nm is observed, which is due to the presence of unavoidable trace iron impurities in the raw materials for glass preparation [17]. The optical absorption spectra of glasses containing 2.5 and 5 mol% WO_3_ display one symmetrical band at 250–260 nm that can be assigned to the ligand–metal charge transfer (LMCT) from oxygen ligands to W^6+^ of distorted and isolated WO_4_ tetrahedra. It is well-known that DRS spectra for the isolated WO_4_ reference compounds only possess a single ligand-to-metal charge transfer (LMCT) band in the general region of 218–274 nm [18]. The exact location of this band maximum depends on the extent of distortion of the isolated WO_4_ structure. For example, K_2_WO_4_ has a relatively undistorted isolated WO_4_ unit and possesses a LMCT band at 223 nm, whereas Zr(WO_4_)_2_ consists of a distorted isolated WO_4_ unit and exhibits an LMCT band at 274 nm. DRS spectra of glasses x = 7.5, x = 10 and x = 20 are broad, and can be regarded as composed of at least two bands—at about 260 nm and at about 285 nm. The UV–Vis diffuse reflectance spectra of various tungstate reference compounds containing octahedrally coordinated W atoms against oxygen show two absorption bands between 250 and 360 nm that can be attributed to octahedral WO_6_ units [18]. Following the above, the bands at 260 nm and at 285 nm in the DRS spectra of glasses containing from 7.5 to 20 mol% WO_3_ can be linked to the presence of WO_6_ octahedra. The absence of any absorption in the visible range indicates that W^5+^ species do not present in the investigated glasses [19]. Optical band gap values (E_g_) evaluated from the UV–Vis spectra can give information about the structural arrangement of the glasses under investigation (inset of Figure 3). The plot of transformed Kubelka–Munk function versus the energy of light (Tauc plot) provides band gap energies, E_g_, listed in Table 1. E_g_ values established for glasses x = 2.5 and x = 5 are typical for band gap energies for tungstates containing WO_4_ tetrahedra [20,21]. The band gap energy values of glasses x = 7.5, x = 10 and x = 20 are compatible with the values of other tungstate glasses possessing octahedrally coordinated W^6+^ (WO_6_) [22]. The decreasing E_g_ with increasing WO_3_ observed suggests that progressive polymerization of WO_6_ groups, i.e., increasing number of bridging W–O–W bonds, takes place upon WO_3_ loading [18]. From DRS results it is seen that there is a strong overlapping of the LMCT band regions, as well as of the band gap energy values for WO_4_ and WO_6_ groups. That is why it is reasonable to suggest that both WO_4_ and WO_6_ polyhedra participate in the structure of glasses having higher WO_3_ concentration (7.5, 10 and 20 mol%).

### 2.3. Density, Molar Volume, Oxygen Packing Density and Oxygen Molar Volume

The influence of WO_3_ on several physical parameters of the investigated glasses such as density (ρ), molar volume (V_m_), oxygen molar volume (V_o_), and oxygen packing density (OPD) has been studied and the data obtained are listed in Table 1. Determination of these physical parameters is the simplest way of detecting structural changes in glass networks with compositional variation. As can be seen from the table for the investigated glasses, both density and oxygen molar values increase with increasing WO_3_ concentration from 2.5 to 20 mol% (Table 1). The density enhancement can be attributed to the increase in the average molecular mass of glasses as a result of the substitution of lighter B_2_O_3_ (molecular weight 69.62 g/mol) and SiO_2_ (molecular weight 60.08 g/mol) with the heavier WO_3_ (molecular weight 231.84 g/mol) [23]. As the molar volume (volume occupied by a mass of the glass equal to 1 mole) is strongly affected by the ionic radii of the incorporated ionic species in the glass, the increasing trend of V_m_ is due to the insertion of W^6+^ ions, which are known to have a higher ionic radius (0.60 Å) compared with the ionic radii of B^3+^ (0.23 Å) and Si^4+^ (0.26 Å), resulting in the formation of an excess free volume, which increases the overall molar volume of glasses [24,25,26].

Oxygen molar volume (V_o_) and OPD provide insight into how oxygen ions are packed within the glass structure [27]. Lower V_o_ and higher OPD values typically indicate a more tightly connected network. V_o_ increases and OPD decreases for glasses with up to 5 mol% WO_3_ (samples x = 2.5 and x = 5) compared to WO_3_-free glass, implying that WO_3_ addition from 2.5 to 5 mol% increases the concentration of non-bridging oxygens (NBOs), resulting in the formation of less packed and more disordered glass network. Glasses with higher WO_3_ concentration (7.5; 10 and 20 mol%) show a decreasing V_o_ and increasing OPD upon WO_3_ loading. Considering the present DRS data, we explain the lowering V_o_ and enhancing OPD values of x = 7.5, x = 10 and x = 20 glasses with the increase in the bridging W–O–W bonds concentration as a result of the accumulation of WO_6_ units and their gradual polymerization [18].

### 2.4. Photoluminescent Properties

**Figure 4:**
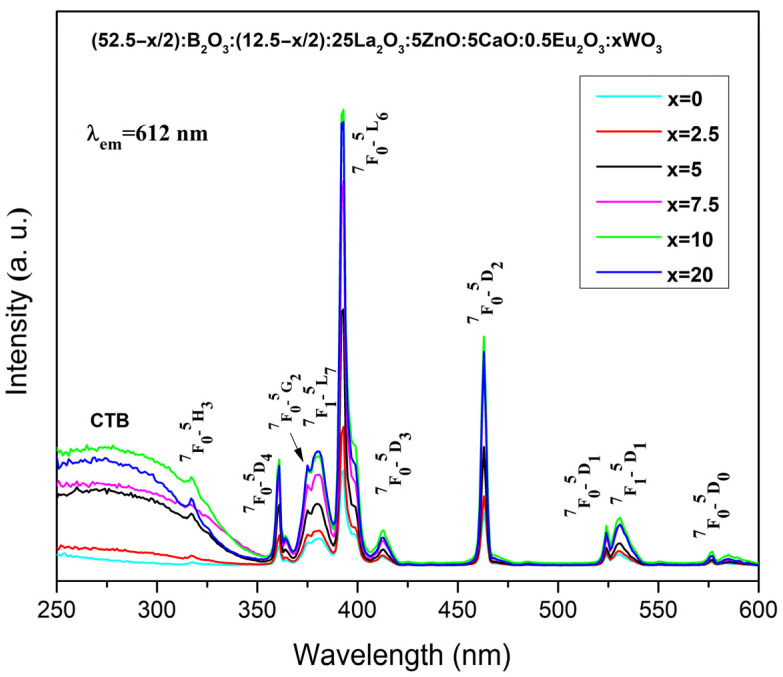
Excitation spectra of investigated Eu^3+^-doped borosilicate glasses.

The excitation spectra of the prepared Eu^3+^-doped glasses are shown in Figure 4. All data were obtained at room temperature by monitoring the most intensive characteristic emission of Eu^3+^ ions at 612 nm wavelength, corresponding to ^5^D_0_ → ^7^F_2_ transition. As can be seen from the figure, a broad, continuous band below 350 nm is observed, along with several narrow peaks distributed across the 350–600 nm wavelength range. Generally, the broadband is due to ligand to metal charge transfer transitions (LMCT) from oxygen 2p orbital to the empty 4f orbital of europium (O^2−^ → Eu^3+^) and from near band-edge (NBE) transition inside the ZnO_n_ (ZnO_n_ = ZnO_4_) host absorbing groups [28,29,30,31,32]. Additionally, in the glasses containing tungsten oxide ((52.5 − x/2)B_2_O_3_:(12.5 − x/2)SiO_2_:25La_2_O_3_:5ZnO:5CaO:0.5Eu_2_O_3_:xWO_3_, x = 2.5, 5, 7.5, 10 and 20 mol%) this band is also due to the transition from O^2−^ → W^6+^ inside the WO_n_ (WO_n_ = WO_4_ and WO_6_) groups [33].

The presence of the excitation band of WO_n_ recorded at the emission wavelength of Eu^3+^ at 612 nm suggests that the energy absorbed by the tungstate groups is subsequently non-radiatively transferred to Eu^3+^ ions [33,34]. As can be observed from Figure 4, with the increase in WO_3_ content the intensity of charge transfer absorption band is rising and the glass containing 10 mol% WO_3_ exhibits highest intensity. Thus, it can be assumed that the more energy is absorbed by WO_n_ groups, the more energy is expected to be transferred to the Eu^3+^, leading to stronger emission. This mechanism is commonly referred to as host-sensitized luminescence. Structural changes are most likely the cause of the slight decrease in the excitation intensity of glass composition with 20 mol% WO_3_.

The distinct sharp peaks observed in the region above 350 nm are attributed to 4f–4f electron transitions from the ground state to excited energy levels, specifically, ^7^F_0_ → ^5^H_3_ (~318 nm), ^7^F_0_ → ^5^D_4_ (~360 nm), ^7^F_0_ → ^5^G_2_ (~376 nm), ^7^F_1_ → ^5^L_7_ (~381 nm), ^7^F_0_ → ^5^L_6_ (~393 nm), ^7^F_0_ → ^5^D_3_ (~413 nm), ^7^F_0_ → ^5^D_2_ (~463 nm), ^7^F_0_ → ^5^D_1_ (~523 nm) and ^7^F_1_ → ^5^D_1_ (~531 nm), and ^7^F_0_ → ^5^D_0_ (~576 nm). The highest excitation was observed at ^7^F_0_ → ^5^L_6_ (393 nm). Therefore, the emission spectra measurements were conducted under excitation at 393 nm. The comparison between the LMCT band and the 4f–4f transitions reveals that the narrow Eu^3+^ peaks exhibit higher intensity. This means that the efficient excitation by near-UV and blue LED chips can be obtained, since Eu^3+^ 4f–4f transitions are typically weak because they are partially forbidden by Laporte’s selection rule [35].

**Figure 5:**
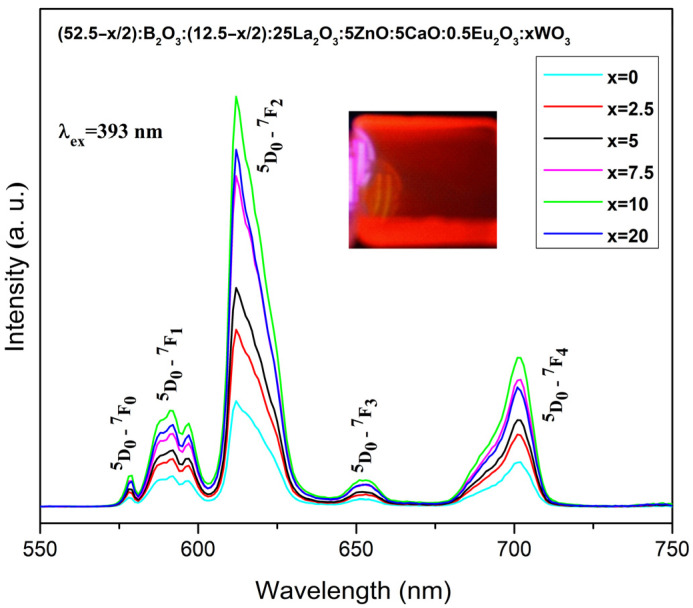
Emission spectra of the investigated Eu^3+^-doped borosilicate glasses and photograph (inset) of the red glow glass under UV exposure.

The emission spectra of Eu^3+^-doped borosilicate glasses (Figure 5) were recorded at room temperature using a 393 nm excitation wavelength. The five narrow characteristic emission peaks, originating from the radiative transitions of Eu^3+^ ions from the ^5^D_0_ excited state to the lower-lying ^7^F_0_, ^7^F_1_, ^7^F_2_, ^7^F_3_, ^7^F_4_ ground states, are observed at 578 nm, 591 nm, 612 nm, 652 nm and 701 nm [36]. Among these bands, the strongest one at 612 nm is due to the ^5^D_0_ → ^7^F_2_ transition, which is responsible for the red emission of the studied dopant. As shown in Figure 5, the emission intensity exhibits a strong dependence on the composition and increases with the incorporation and rising of tungsten content in the host matrix. This behavior can be related to non-radiative energy transfer between the glass host and the luminescent Eu^3+^ ions. The glass with 10 mol% WO_3_ is characterized with the highest emission, which is in accordance with the already established highest excitation intensity of the same composition shown in Figure 4. The glass containing 20 mol% WO_3_ transfers less energy to the active ion due to their less intense excitation.

Additional evidence for this energy-transfer mechanism is provided by the absence of the typical broad WO_3_ emission band in the 400–600 nm [37,38] spectral region, indicating that the excitation energy absorbed by tungstate groups is efficiently transferred to the Eu^3+^ ions.

Among all the observed emission bands, the most intense one, centered at 612 nm, originates from the forced electric-dipole (ED) ^5^D_0_ → ^7^F_2_ transition, which is highly sensitive of the local crystal-field environment surrounding the Eu^3+^ ions, followed by the magnetic-dipole (MD) ^5^D_0_ → ^7^F_1_ at 591 nm transition, which is insensitive to the surrounding ligands [28,36]. The dominance of the ED transition over the MD transition indicates that Eu^3+^ ions occupy non-centrosymmetric sites within the glass host. Moreover, the ratio of these emission intensities, commonly referred to the asymmetry ratio R = (^5^D_0_ → ^7^F_2_)/(^5^D_0_ → ^7^F_1_), provides insight into the degree of local asymmetry surrounding around the Eu^3+^ ions, as well as the strength of Eu–O covalence in various Eu^3+^-doped materials [39,40]. Lower values of the asymmetry parameter correspond to higher local site symmetry around the active ion and lower Eu–O covalency and emission intensity. The increase in R value is due to the increase in asymmetry and covalency between the Eu^3+^ ion and the ligands and leads to a higher emission intensity [41]. The R values of the synthesized glasses are listed in Table 2, along with other data reported in the literature for Eu^3+^-doped glasses and the commercial phosphor material used.

Compared to our previously synthesized glasses containing boron and tungsten oxides, the values of the asymmetric ratio R are similar [16,42,43,44], but compared to other borate or silicate oxide glass compositions [45,46,47,48,49,50,51] the values are higher. The relatively higher R values observed in the present glasses indicate that Eu^3+^ ions occupy low-symmetry sites and provide evidence of the high Eu^3+^-O^2−^ covalency. The highest asymmetry ratio is obtained for glass compositions with 10 and 20 mo % WO_3_ that show the highest emission intensity. In addition, the ^5^D_0_ → ^7^F_1_ transition of Eu^3+^ exhibits splitting into three emission peaks. This behavior is attributed to crystal-field-induced splitting, where a single electronic transition gives rise to multiple emission components [52]. Moreover, the presence of the ^5^D_0_ → ^7^F_0_ transition, which is highly sensitive to the local crystal field and normally forbidden under standard Judd–Ofelt theory, further confirms that Eu^3+^ ions reside at non-centrosymmetric sites with C_2v_, C_n_, or C_s_ symmetry within the glass matrix [53].

**Figure 6:**
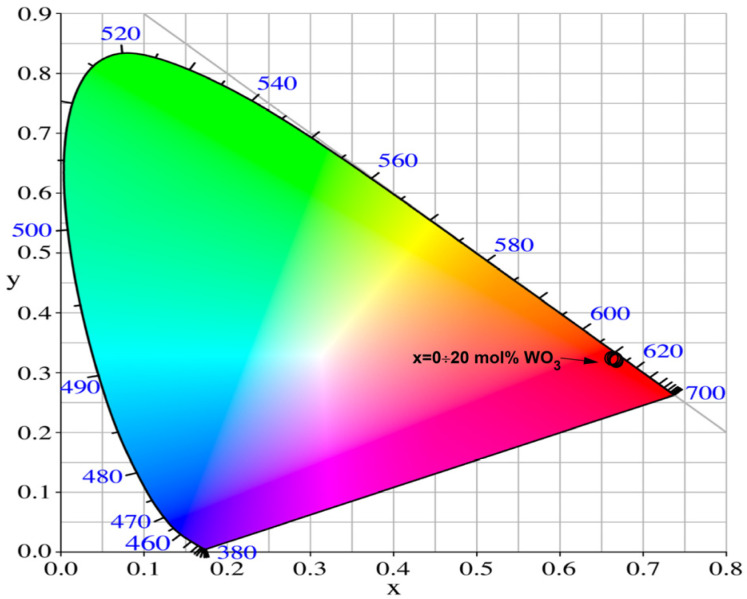
CIE chromaticity diagram of investigated Eu^3+^-doped borosilicate glasses.

Furthermore, to evaluate the luminescent properties and the perceived emission color, the Commission Internationale de l’Éclairage (CIE) 1931 chromaticity diagram was used [54]. The chromaticity coordinates of the synthesized borosilicate glasses were calculated from the photoluminescence spectra (Figure 5) using SpectraChroma software (Version 1.0.1, CIE coordinate calculator) [55]. The obtained values (Table 3) are almost identical and cannot be individually separated on the CIE diagram. They are located in the red region in Figure 6. The calculated coordinates are very close to the NTSC standard for red light (0.670, 0.330), as well as to the chromaticity coordinates of the commercial red phosphor Y_2_O_2_S:Eu^3+^ (0.658, 0.340) [56].

Based on the performed analyses, it was found that various factors influence the emission intensity of Eu^3+^ ions. On the one hand, we established the influence of tungsten oxide, i.e., increasing the emission by implementing a non-radiative energy transition from tungsten to the luminescent active ion. On the other hand, WO_3_ affects the structure of the host matrix. More asymmetric site positions are created in the presence of WO_3_ in which Eu^3+^ ions can be incorporated. The asymmetric positions of Eu^3+^ ions are beneficial for its luminescent properties. In the glass network, WO_4_ and WO_6_ units participate simultaneously. At low tungsten content, mainly WO_4_ units are present, while WO_4_ tetrahedra and WO_6_ octahedra co-exist with increasing WO_3_ content. The optimal concentration at which the highest emission intensity is observed is 10 mol% WO_3_, due to the suitable ratio of WO_4_/WO_6_ ensuring the highest excitation intensity and Eu^3+^ sites with the highest asymmetry. The lower excitation and emission intensity is observed in the glass containing 20 mol% WO_3_ with a higher content of WO_6_ units.

## 3. Materials and Methods

Several glass samples of (52.5 − x/2)B_2_O_3_:(12.5 − x/2)SiO_2_:25La_2_O_3_:5ZnO:5CaO:0.5Eu_2_O_3_:xWO_3_, (x = 0, 2.5, 5, 7.5, 10 and 20 mol%) compositions were obtained by applying the melt quenching method, using reagent-grade La_2_O_3_, H_3_BO_3_, SiO_2_, CaO, ZnO, Eu_2_O_3_ and WO_3_ as raw materials. The homogenized batches (5 g) were melted at 1400 °C for 2 h in corundum crucibles in air. The melts were cast into a preheated graphite mold to get bulk glass samples. Then, the glasses were transferred into a laboratory electric furnace, annealed at 500 °C for 1 h, and cooled down to room temperature at a very slow cooling rate of about 0.5 °C/min. The phase formation of the samples was established by X-ray phase analysis with a Bruker (Karlsruhe, Germany) D8 Advance diffractometer, using Cu Kα radiation in the 10 < 2θ < 60 range. The glass transition (T_g_) temperatures of the glasses were determined by differential scanning calorimetry (DSC) using a Netzsch (Selb, Germany) 404 Pegasus instrument, 2021 Selb, Germany, at a heating rate of 10 K/min in an Ar flow of 10 mL/s, using corundum crucibles with lids. The density of the obtained glasses at room temperature was measured by the Archimedes principle using toluene (ρ = 0.867 g/cm^3^) as an immersion liquid on a Mettler Toledo electronic balance of sensitivity 10^−4^ g. From the experimentally evaluated density values, the molar volume (V_m_), the molar volume of oxygen (V_o_) (volume of glass in which 1 mol of oxygen is contained) and the oxygen packing density (OPD) of the glasses obtained were estimated, using the following relations respectively:

(1) $$V_{m}=\frac{\sum x_{i}M_{i}}{\rho_{g}}$$

(2) $$V_{o}=V_{m}\;\times \;\left(\frac{1}{\sum x_{i}n_{i}}\right)$$

(3) $$OPD=1000\;\times \;C\;\times \;\left(\frac{\rho_{g}}{M}\right)$$

where ***x_i_*** is the molar fraction of each component i, ***M_i_*** is the molecular weight, ***ρ_g_*** is the glass density and ***n_i_*** the number of oxygen atoms in each oxide, ***C*** is the number of oxygen per formula units, and ***M*** is the total molecular weight of the glass compositions. The optical spectra of the powder samples at room temperature were recorded with a spectrometer (Evolution 300 UV–vis Spectrophotometer, London, UK) employing the integration sphere diffuse reflectance attachment. The samples were measured in the wavelength (λ) range of 200–1100 nm with a magnesium oxide reflectance standard used as the baseline. The uncertainty in the observed wavelength is about ±1 nm. The Kubelka–Munk function (F(R∞)) was calculated from the UV–Vis diffuse reflectance spectra. The band gap energy (E_g_) was determined by plot (F(R∞) hν)^1/n^, n = 2 versus hν (incident photon energy). Photoluminescence (PL) excitation and emission spectra at room temperature for all glasses were measured with a Spectrofluorometer FluoroLog3-22, 2014 (Horiba JobinYvon, Longjumeau, France).

## 4. Conclusions

In this study, the influence of WO_3_ on the physical parameters and luminescent characteristics of Eu^3+^-doped complex borosilicate glasses (52.5 − x/2)B_2_O_3_:(12.5 − x/2)SiO_2_:25La_2_O_3_:5ZnO:5CaO:0.5Eu_2_O_3_:xWO_3_, x = 0, 2.5, 5, 7.5, 10, 20 (mol%) was established. The XRD results confirmed the amorphous nature of the glasses. The optical absorption spectra contained major bands corresponding to W^6 +^ ions in tetrahedral and octahedral positions (WO_4_ and WO_6_). W-O-W bonds were also formed in the WO_3_-containing glasses with between 7.5 and 20 mol% WO_3_. The densities were in the range of 3.792–4.607 g/cm^3^. The positive effect of WO_3_ on the luminescence intensity of the Eu^3+^-doped complex borosilicate glass was established. It was found that the introduction of WO_3_ in the borosilicate glass network ensures a more asymmetrical local structure around Eu^3+^ sites, accordingly yielding a higher luminescence of the incorporated Eu^3+^ ions. On the other hand, tungsten oxide has a synthesizer effect by transferring the emission energy non-radiatively to the activator Eu^3+^, which additionally improves its luminesce properties. It was established that the optimum WO_3_ concentration to obtain the most intensive red luminescence is 10 mol%, making this glass sample a suitable candidate for visible red emission applications.

## Figures and Tables

**Table 1:** Values of physical parameters of glasses (52.5 − x/2)B_2_O_3_:(12.5 − x/2)SiO_2_:25La_2_O_3_:5ZnO:5CaO:0.5Eu_2_O_3_:xWO_3_, x = 0, 2.5, 5, 7.5, 10 and 20 mol%: density (ρ_g_), molar volume (V_m_), oxygen molar volume (V_o_), oxygen packing density (OPD), glass transition temperature (T_g_) and optical band gap (E_g_).

Sample ID	ρ_g_ (g/cm^3^)	V_m_ (cm^3^/mol)	V_o_ (cm^3^/mol)	OPD (g atom/L)	T_g_ (°C)	E_g_ (eV)
x = 0	3.792 ± 0.002	35.38	13.15	76.03	555; 663	3.54
x = 2.5	3.892 ± 0.002	35.54	13.16	75.97	601; 675	4.04
x = 5	3.973 ± 0.004	35.87	13.21	75.69	684	3.52
x = 7.5	4.088 ± 0.002	35.88	13.14	76.09	682	3.84
x = 10	4.177 ± 0.001	36.11	13.13	76.16	671	3.79
x = 20	4.607 ± 0.001	36.34	13.03	76.77	647	3.70

**Table 2:** Comparison of intensity ratio R of Eu^3+^-doped borosilicate glasses with different host matrices.

Glass Composition	Relative Intensity Ratio, R	Reference
52.5B_2_O_3_:25La_2_O_3_:12.5SiO_2_:5CaO:5ZnO:0.5Eu_2_O_3_	4.12	Current work
51.25B_2_O_3_:11.25SiO_2_:25La_2_O_3_:5CaO:5ZnO:2.5WO_3_:0.5Eu_2_O_3_	4.33	Current work
50B_2_O_3_:25La_2_O_3_:10SiO_2_:5CaO:5ZnO:5WO_3_:0.5Eu_2_O_3_	4.42	Current work
48.75B_2_O_3_:8.75SiO_2_:25La_2_O_3_:5CaO:5ZnO:7.5WO_3_:0.5Eu_2_O_3_	4.61	Current work
47.5B_2_O_3_:7.5SiO_2_:25La_2_O_3_:5CaO:5ZnO:10WO_3_: 0.5Eu_2_O_3_	4.86	Current work
42.5B_2_O_3_:2.5SiO_2_:25La_2_O_3_:5CaO:5ZnO:20WO_3_: 0.5Eu_2_O_3_	4.92	Current work
50ZnO:(49 − x)B_2_O_3_:1Bi_2_O_3_:xWO_3_: 0.5Eu_2_O_3_ x = 1, 5, 10,	4.61–5.73	42
50ZnO:40B_2_O_3_:10WO_3_:xEu_2_O_3_ (0 ≤ x ≤ 10)	4.54–5.77	43
50ZnO:(50 − x)B_2_O_3_:xNb_2_O_5_:0.5Eu_2_O_3_:, x = 0, 1, 3 and 5 mol%	4.31–5.16	44
50ZnO:(50 − x)B_2_O_3_:0.5Eu_2_O_3_:xWO_3_, x = 0, 1, 3, 5.	4.34–5.57	16
89.5B_2_O_3_–10Li_2_O–0.5Eu_2_O_3_	2.41	45
64SiO_2_-16K_2_O-16BaO-4Eu_2_O_3_	3.42	
0.5GeO_2_-63.5SiO_2_-16K_2_O-16BaO-4Eu_2_O_3_	3.46	
4ZnO:3B_2_O_3_ 0.5–2.5 mol% Eu_2_O_3_	2.74–3.94	46
60ZnO:20B_2_O_3_:(20 − x)SiO_2_ − xEu_2_O_3_ (x = 0 and 1)	3.166	47
74.5B_2_O_3_ + 10SiO_2_ + 5 MgO + 5x + 0.5 Eu_2_O_3,_ x = Li_2_O + Na_2_O; Li_2_O + K_2_O and K_2_O + Na_2_O	2.102–2.266	1
20 *M*F_2_·69 B_2_O_3_·10 Al_2_O_3_·1Eu_2_O_3,_ *M* = Ca, Pb and Zn	3.77–5.89	48
35B_2_O_3_–20SiO_2_-(15 − x) Al_2_O_3_ − 15ZnO-15Na_2_CO_3_-xEu_2_O_3_ (x = 0.0, 0.5, 1.0, 1.5, 2.0, 2.5 mol%)	3.62–3.92	6
50B_2_O_3_-19SiO_2_-20Na_2_O-10CaO-1Eu_2_O_3_	3.151	49
50B_2_O_3_-14SiO_2_-20Na_2_O-10CaO-5ZnO-1Eu_2_O_3_	3.352	
50B_2_O_3_-14SiO_2_-20Na_2_O-10CaO-5TeO_2_-1Eu_2_O_3_	4.269	
Eu^3+^:Y_2_O_3_	3.8–5.2	50, 51

**Table 3:** CIE chromaticity coordinates of the borosilicate glasses.

Glass Composition	Chromaticity Coordinates (x, y)
52.5B_2_O_3_:25La_2_O_3_:12.5SiO_2_:5CaO:5ZnO:0.5Eu_2_O_3_	0.649, 0.348
51.25B_2_O_3_:11.25SiO_2_:25La_2_O_3_:5CaO:5ZnO:2.5WO_3_:0.5Eu_2_O_3_	0.652; 0.348
50B_2_O_3_:25La_2_O_3_:10SiO_2_:5CaO:5ZnO:5WO_3_:0.5Eu_2_O_3_	0.652, 0.348
48.75B_2_O_3_:8.75SiO_2_:25La_2_O_3_:5CaO:5ZnO:7.5WO_3_:0.5Eu_2_O_3_	0.655; 0.344
47.5B_2_O_3_:7.5SiO_2_:25La_2_O_3_:5CaO:5ZnO:10WO_3_: 0.5Eu_2_O_3_	0.654; 0.346
42.5B_2_O_3_:2.5SiO_2_:25La_2_O_3_:5CaO:5ZnO:20WO_3_: 0.5Eu_2_O_3_	0.654; 0.346
NTSC standard for red light	0.670, 0.330
Y_2_O_2_S:Eu^3+^	0.658, 0.340

## Data Availability

Data are contained within the article. Further inquiries can be directed to the corresponding author.
